# Current research on fungi in chronic wounds

**DOI:** 10.3389/fmolb.2022.1057766

**Published:** 2023-01-11

**Authors:** Yumei Ge, Qingqing Wang

**Affiliations:** ^1^ Department of Clinical Laboratory, Laboratory Medicine Center, Zhejiang Provincial People’s Hospital (Affiliated People’s Hospital, Hangzhou Medical College), Hangzhou, China; ^2^ Institute of Immunology, Zhejiang University, Hangzhou, China; ^3^ The Key Laboratory for Immunity and Inflammatory Diseases of Zhejiang Province, Hangzhou, China; ^4^ The Key Laboratory of Biomarkers and In Vitro Diagnosis Translation of Zhejiang province, Hangzhou, China

**Keywords:** drug-resistant, bacteria, fungi, chronic wounds, diagnostic, therapy

## Abstract

The occurrence of chronic wounds is a major global health issue. These wounds are difficult to heal as a result of disordered healing mechanisms. The most common types of chronic wounds are diabetic ulcers, pressure ulcers, arterial/venous ulcers and nonhealing surgical wounds. Although bacteria are an important cause of chronic nonhealing wounds, fungi also play a substantial role in them. The fungal infection rate varies with different chronic wound types, but overall, the prevalence of fungi is extremely underestimated in the clinical treatment and management of chronic wounds. Wounds and ulcers can be colonized by host cutaneous, commensal or environmental fungi and evolve into local infections, causing fungemia as well as invasive fungal disease. Furthermore, the fungi involved in nonhealing wound-related infections help commensal bacteria resist antibiotics and the host immune response, forcing wounds to become reservoirs for multiresistant species, which are considered a potential key factor in the microbial bioburden of wounds and ulcers. Fungi can be recalcitrant to the healing process. Biofilm establishment is the predominant mechanism of fungal resistance or tolerance to antimicrobials in chronic nonhealing wounds. *Candida albicans* yeast and *Trichophyton rubrum* filamentous fungi are the main fungi involved in chronic wound infection. Fungal species diversity and drug resistance phenotypes in different chronic nonhealing wound types will be emphasized. In this review, we outline the latest research on fungi in chronic wounds and discuss challenges and future perspectives related to diagnosing and managing chronic wounds.

## Introduction

Chronic wounds are defined as wounds not healing at the expected rate for a long duration (>6 weeks) with a lack of functional restoration after 3 months (Fonder, et al., 2008). Compared with acute wounds, chronic wounds present the characteristics of delayed healing or even non-healing. The incidence of chronic wounds is approximately 1%–4% (Rahim, et al., 2017). The most common types of chronic wounds are diabetic ulcers, pressure ulcers, arterial/venous ulcers, burn wounds and non-healing surgical wounds. Chronic wounds may also be the result of other uncommon causes, for example, inflammatory processes (vasculitis, autoimmune diseases, pyoderma gangrenosum), aging, chronic infections, cardiovascular diseases and neoplasms. Malnourishment and chronic mechanical stress have also been confirmed as main predispositions to poor wound healing (Tomic-Canic, et al., 2020). Wound healing consists of four phases: coagulation, inflammation, proliferation and wound remodeling (Reinke, et al., 2012; Morton, et al., 2016). Chronic wounds stagnate in the inflammatory phase (Velnar, et al., 2009). Chronic wounds are believed to be colonized by polymicrobial communities containing bacteria and fungi. Polymicrobial interactions during wound infections contribute to continued inflammation and delayed healing (Dalton, et al., 2011; Rhoads, et al., 2012; Percival, et al., 2015). The production of biofilms by polymicrobial communities contributes to infection tolerance to antibiotic therapy (Wong, et al., 2022) and delayed healing of chronic wounds (Bjarnsholt, et al., 2008; James, et al., 2008; Fan, et al., 2009). The occurrence of chronic wounds is a major global health issue affecting millions of patients worldwide and cause an enormous economic burden on healthcare systems (Haalboom, 2018). According to the retrospective analysis of Medicare data, costs for chronic non-healing wounds are estimated from 28.1 to 96.8 billion dollars (Nussbaum et al., 2018). The Wound Care Market accounts for approximately 18.22 billion dollars and is estimated to reach 26.24 billion dollars worldwide in 2023 according to the latest Global Wound Care Market report (Weller et al., 2020). More than 38 million cases of chronic wound infections occur due to medical shortcomings in wound healing and are associated with poor disease prognoses (Nosrati, et al., 2021). Chronic wounds can increase comorbidity and mortality rates in elderly patients (Mihai, et al., 2018). For example, diabetic foot ulcers contribute to 80% of nontraumatic lower-extremity amputations, and their 5-year mortality rate is approximately 43%–55%, which is higher than those of breast cancer, prostate cancer and Hodgkin’s disease (Boulton, et al., 2005; Armstrong, et al., 2007; Robbins, et al., 2008).

Persistent infection, prolonged inflammation and biofilms composed of various microorganisms that are resistant to the host immune response and antibiotics are common features of chronic wounds. Microorganisms have become a focus in complex wound healing in recent years. Wounds and ulcers provide favorable conditions for pathogenic microorganisms from the skin microbiota and environment to invade deep tissues and find optimal conditions for colonization and growth (Zeeuwen, et al., 2012). Wound microbiomes formed by bacterial and fungal colonization are thought to stall healing and cause chronicity by community microbial processes (Tipton, et al., 2020). Little mention has been made concerning the contribution of fungi to chronic wound-related infections, and the prevalence of fungi, including *Candida spp*. and *Aspergillus* spp. is extremely underestimated in the clinical treatment and management of chronic wounds. It has been demonstrated that fungi play a significant role as opportunistic and primarily pathogenic components in chronic wounds. Wounds and ulcers can be colonized by host cutaneous, commensal or environmental fungi and evolve into local infections, causing fungemia as well as invasive fungal disease. Furthermore, the fungi involved in non-healing wound-related infections help commensal bacteria resist antibiotics and the host immune response, forcing wounds to become reservoirs for multiresistant species, which are considered a potential key factor in the microbial bioburden of wounds and ulcers. Chronic non-healing wound type, duration, location and stages, and patient topographical and temporal diversity all influence the resistant phenotypes, ascendency and mixture of fungal species. It has been reported that 23% of 915 chronic wounds, including diabetic foot ulcers, pressure ulcers, non-healing surgical wounds and venous ulcers, were positive for fungal species. Although yeast species in the genus *Candida* have the highest incidence rate, nonconventional fungi, including *Curvularia*, *Malessezia*, *Aureobasidium*, *Cladosporium*, *Ulocladium*, *Engodontium* and *Trichophyton,* are also prevalent in the chronic wound bioburden (Dowd, et al., 2011). The prevalence of diabetic wounds infected with fungi is 9%–40.1%, of which the predominant fungal species are *Candida albicans*, *Candida tropicalis*, *Candida parapsilosis* and *Candida guilermondii*, followed by *Aspergillus flavus*, *Aspergillus niger* and *Fusarium* spp. (Bansal, et al., 2008). An analysis of fungi prevalence in 152 lower extremity ulcers and the surrounding skin showed that 6% of ulcer samples and 27.6% of skin samples were positive for three fungal species, *Candida albicans*, *Candida parapsilosis* and *Candida ciferrii* (Foroozan, et al., 2011). An analysis of pathogens from 1,310 thermal burn wounds showed that 110 (5.04%) fungal strains were isolated with the prevalent constituent mixture of *Candida parapsilosis*, *Aspergillus flavus*, *Candida albicans* and *Candida tropicalis*, and all *Candida albicans* were susceptible to voriconazole, amphotericin B, fluconazole, itraconazole, and ketoconazole treatment (Zhang, et al., 2018). Fungal wound infections pose a special challenge and cause substantial morbidity among burn patients (Ladhani, et al., 2021). The negative consequences of these fungi cause large healthcare burdens, including chronic wounds or nonhealing wounds (Sen, et al., 2009; Guariguata, et al., 2014; Kerr, et al., 2014). The current understanding of resistant phenotypes, relative abundance and species diversity of fungi, occurrence, and tendency of cohabitation of fungal and bacterial species in chronic wounds should be further elucidated. This review briefly discusses the wound healing process and impaired healing in chronic wounds, the latest research on fungi in chronic wounds, and diagnostic and therapeutic innovations for chronic wound care and highlights challenges and future perspectives related to diagnosing and managing chronic wounds.

## Wound healing process and impaired healing in chronic wounds

Coagulation, inflammation, proliferation and wound remodeling constitute the well-organized wound healing process (Sun, et al., 2014; Morton, et al., 2016). Immediately after injury, coagulation starts to prevent bleeding and form a blood clot (Demidova-Rice, et al., 2012). The blood clot protects the wound and allows the migration of leukocytes, fibroblasts and keratinocytes (Morton, et al., 2016). The blood clot is trapped by blood platelets and has cytokines and growth factors that can attract neutrophils, endothelial cells, macrophages and fibroblasts in the inflammatory and proliferation phases (Velnar, et al., 2009). Neutrophils are immediately attracted by growth factors and cytokines during the coagulation phase. In the inflammation phase, the central goal is destruction and removal of bacteria (Tsourdi, et al., 2013). Macrophages and lymphocytes support the inflammatory response, and inflammatory cells are vital in the defense against bacteria and are the source of many cytokines and growth factors. These cells can initiate the proliferation phase (Werner, et al., 2003). The development of granulation tissue is a feature of the proliferation phase (Tsourdi, et al., 2013). In the wound area, fibroblasts migrate and combine various components. Moreover, fibroblasts differentiate into myofibroblasts, which play major roles in wound edges at the end of the proliferation phase (Werner, et al., 2003). These phases result in the formation of new blood vessels to supply adequate blood. Vascular endothelial growth factor (VEGF), platelet-derived growth factor (PDGF) and fibroblast growth factor are produced when blood vessels are damaged during injury (Demidova-Rice, et al., 2012; Sun, et al., 2014). These factors secrete proteolytic enzymes that enable endothelial cells to enter the proliferation phase and migrate into wound tissues (Reinke, et al., 2012). The last process of wound healing is remodeling. The extracellular matrix, which is composed of fibroblasts, is degraded by matrix metalloproteinase enzymes (MMPs). Stronger collagen I will replace it to form a scar. Myofibroblasts can facilitate wound contraction to close wound tissues (Morton, et al., 2016). Then, fibroblasts are eliminated, and angiogenesis is inhibited. Finally, the wound healing process is complete after the remodeling phase.

Due to an imbalance between inflammatory cells and inhibitors, chronic wounds usually stagnate in the inflammatory phase (Schultz, et al., 2003; Satish, 2015). Overexpression of interferon-gamma (IFN-γ), a proinflammatory cytokine that is essential for the regulation of collagen synthesis, delays wound healing, and dysfunction of interleukin-17 (IL-17) inhibits progression to the proliferation phase in nonhealing wounds (Abedini, et al., 2022). Compared with acute wounds, nonhealing wounds display clinical, morphological, microbiological and biochemical differences (Kirker, et al., 2017). Internal wound pressure, which occurs in conditions such as venous insufficiency, external pressure, such as that observed in diabetic neuropathy, bacterial contamination and repeated ischemic injury, results in continuous inflammation in wound beds (Mast, et al., 1996). Inflammatory cells secrete cytokines and can produce proteolytic enzymes such as MMPs. MMPs can degrade components of the extracellular matrix, growth factors and growth factor receptors, which are vital for wound healing (Menke, et al., 2007). Abnormally increased interleukin-10 (IL-10) in diabetic chronic wounds causes significant reductions in Toll-like receptor signaling and proinflammatory cytokine production, which underlies healing impairment in diabetic wounds (Roy, et al., 2022). In the proliferation phase, fibroblasts are useful for decreasing growth factor receptors and reducing migration in chronic wounds, inhibiting the formation of granulation tissue and stimulating remodeling. However, chronic wound fibroblasts are less responsive to transforming growth factor beta (TGF-β) and PDGF (Mihai, et al., 2018). Chronic wounds are characterized by a highly inflammatory microenvironment with an overabundance of opportunistic pathogens, proinflammatory macrophages, inflammatory mediators such as tumor necrosis factor-α and interleukin-1β, matrix metalloproteinases and reactive oxygen species, leading to a vicious cycle of biofilm formation, continuous inflammation and delayed healing (Raziyeva, et al., 2021). Therefore, the proliferation phase of chronic wounds is impaired (Satish, 2015). Chronic wound cells have lower multiplying rates and are similar to senescent cells, leading to the prolonged presence of neutrophils and proinflammatory macrophages. Chronic wounds can also promote inflammation and tissue fibrosis and reduce angiogenesis, causing hypoxia due to a low blood supply.

Different types of chronic wounds have different pathophysiological processes. Venous ulcers are the result of valvular incompetence and can lead to increases in blood pressure and vessel permeability, resulting in fibrin production and decreased collagen synthesis. Pressure ulcers are caused by pressure applied to the skin and underlying tissue and frequently occur in patients who have decreased mobility and sensory abilities (Demidova-Rice, et al., 2012). Hospitalization and long-term decubitus usually cause pressure ulcers (Rahim, et al., 2017). In addition, insufficient blood flow can cause arterial ulcers. These wounds are frequently triggered by the presence of bacteria, and chronic inflammation is a common feature. During ulcer formation, inflammatory cells produce reactive oxygen species and lead to extracellular matrix damage (Mihai, et al., 2018). Advanced stages of pressure ulcers are polymicrobial and linked with biofilm-associated infection, and phenotypic hypervariability of species, including fungi and inherent resistance to antimicrobials, constitutes a major clinical challenge (Gomes, et al., 2022).

The development of chronic wounds in patients is challenging to decipher. Once these wounds appear, the initial stages have already passed. Therefore, animal models of chronic wounds are of great importance. Dhall et al. created chronic wounds in mice by manipulating impaired wounds in LIGHT^−/−^ mice (Dhall, et al., 2014). Such mouse model wounds can remain open for several weeks and mimic some features of human chronic wounds. LIGHT^−/−^ model mice can show elevated levels of genes involved in nitrosative stress and oxidation, leading to imbalanced redox levels that are exacerbated with age in wound tissues. The redox burden can cause deleterious effects that can impair healing in wound tissues (Dhall, et al., 2014).

**FIGURE 1:**
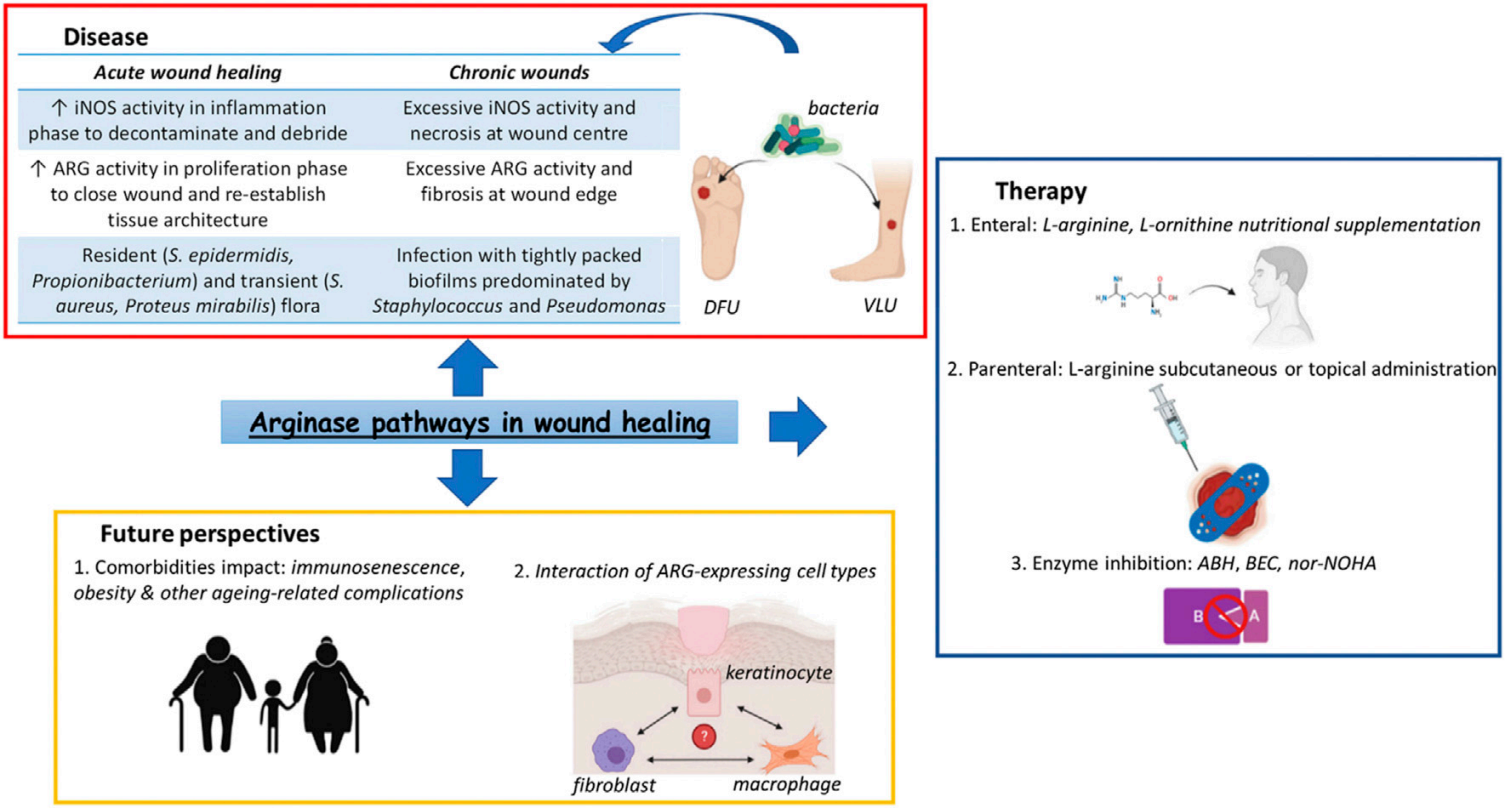
Arginase pathways in wound healing (Szondi, Wong, et al., 2021).

Some enzymes are also important in the wound healing process. Arginase (ARG) is a conserved enzyme that can be expressed by multiple skin cell types (Figure 1) (Szondi, et al., 2021). ARGs can play multiple roles in wound healing, such as balancing pro- and anti-inflammatory responses. In delayed healing wounds, there is defective ARG signaling, expression and function. Therefore, manipulating the ARG pathway is critical to enhance the healing process and improve the outcomes of patients (Szondi, et al., 2021). It was reported that increased fungal chitin exposure induced host ARG activity and that *Candida albicans* blocked nitric oxide production in human macrophages by inducing ARG activity, reducing the ability of macrophages to eradicate the fungus (Wagener, et al., 2017).

## Fungi in chronic wounds

Fungi can cause many substantial wound infections ranging from allergic syndromes to life-threatening invasive fungal diseases (IFDs) (Bongomin, et al., 2017). Many studies have focused on the role of bacterial infection in the wound healing process; however, our skin and environment are also rich in fungi. A previous study showed that approximately 23% of chronic wounds contained fungi (Dowd, et al., 2011). Another study also found fungi in 27% of 518 diabetic lower leg wounds (Chellan, et al., 2010). Seven pathogenic yeasts were isolated from patients with diabetic wounds, with *Candida albicans* being the most common, followed by *Candida lusitaniae* and *Candida dubliniensis*. *Penicillium spp*., *Aspergillus spp*., *Microsporum* spp. and *Trichophyton mentagrophytes* are also significant causes of infections in diabetic foot ulcers (Musyoki, et al., 2022). Five cases of *Aspergillus* chronic subcutaneous infections in patients with diabetes were reported in a meta-analysis of 2,704 fungal isolates from human mycetoma (van de Sande, 2013). The American Burn Association’s Multicenter Trials Group reported that 6.3% of burn patients had positive fungal cultures obtained from the wound itself (Ballard, et al., 2008). Ten fungi species (7%) were collected from 140 wound specimens from Pakistani patients all over the country, among which the highest levels of fungi were isolated from burn and blast wounds. *Candida* species and *Fusarium* species were the most common fungi, indicating that fungal necrotic wound infection is a major challenge in chronic wound management (Farooqi, et al., 2022). When chronic wound infection occurs, the fungal composition of the infection biofilm is an important factor. Chronic nonhealing wounds are hosts to polymicrobial communities. Therefore, they can form biofilms that interfere with healing processes (Kalan, et al., 2016). Fungal infections are also associated with worsening and delaying the healing process of chronic wounds, commonly involving poor clinical outcomes (Mandras, et al., 2022). The most frequently isolated pathogenic fungi and opportunistic pathogenic fungi mainly include *Candida* species and filamentous fungi. However, the biological characteristics, distribution in the human body, secreted invasive enzymes and toxins, drug resistance phenotypes, pathogenic processes and treatment strategies of these two types of fungi are significantly different.

Fungi of the *Candida* genus can be detected in approximately 60% of immunocompetent individuals as symbiotic microbiota (Tanaka, et al., 2021). Some human commensal fungi are opportunistic pathogens and prolific biofilm formers (Ghannoum, et al., 2010; Nunes, et al., 2013). *Staphylococcus* spp. and *Candida* spp. generally predominate the polymicrobial population in chronic and difficult-to-heal wounds (Peng, et al., 2022). The polymicrobial biofilms of bacteria and fungi in burn wound infections have been emphasized, as *Staphylococcus aureus* showed a great adhesion capacity to the surface of *Candida albicans* in biofilms (Hernandez-Cuellar, et al., 2022). *Candida albicans* is one of most important wound fungal pathogens (Singh, et al., 2022), and the key arsenal of *Candida* species is biofilms, a self-sustained pathogenic community that is intrinsically resistant to current antifungal agents (Mishra, et al., 2021). The pathogenicity of *Candida* spp. in chronic wounds is primarily attributed to a variety of virulence factors that help to form biofilms and their escalating resistance to current antifungals, which can invade wounds to form invasive candidiasis or even candidemia, ultimately leading to extremely high mortality rates (Alherz, et al., 2022).

Dermatophyte fungi destroy the stratum corneum and provide an exposed environment for subsequent colonization by common microbes. Filamentous fungi are found around the cellulitis of chronic nonhealing wounds, and angioinvasion of fungal hyphae leads to tissue damage and devastating complications such as mycotic aneurysm, nerve compression, mycotic carbuncle and brain ischemia (Kurokawa, et al., 2022). Compared with yeast-like fungi, the mechanism of filamentous fungi in the process of chronic wound infection is complex. *Trichophyton rubrum* and *Trichophyton mentagrophytes* are the most commonly isolated fungi from patients with onychomycosis in India, and these dermatophytes are likely to be potential factors in chronic wounds (Kayarkatte, et al., 2020). *Trichophyton rubrum* was more abundant in type 2 diabetes patients than in healthy individuals, which indicates that imbalances in the host-microbiota equilibrium caused by proliferation of dermatophytes, followed by biofilm formation, contribute to chronic nonhealing wounds along with peripheral neuropathy and vasculopathy and destruction of local skin immunity in diabetic foot ulcers (Han, et al., 2020). Biofilm establishment is the predominant mechanism of fungal resistance and pathogenicity in many filamentous fungi, such as species belonging to the *Aspergillus*, *Fusarium, Scedosporium*, *Trichophyton*, *Trichosporon* and *Coccidioides* genera. Compared to planktonic-growing conidial cells, the biofilm-forming cells of *Fonsecaea pedrosoi* and *Phialophora verrucosa* are more resistant to conventional antifungal drugs, and their efflux pump activities are higher, which makes their treatment more difficult and even leads to secondary bacterial infection (Sousa, et al., 2022). Patients with shunt devices, chronic rhinosinusitis, keratitis, phaeohyphomycosis, osteomyelitis, diabetic foot ulcers, onychomycosis, traumatic injuries, dermatitis and immunocompromised patients as a result of radiation malignancy and transplant have all been reported to have fungal infections (Montero, et al., 2000; Sampathkumar, et al., 2001; Mendelsohn, et al., 2002; Foster, et al., 2005; Badenoch, et al., 2006; Kaya, et al., 2007; Leake, et al., 2009; Vitrat-Hincky, et al., 2009). Although *Fusarium* spp. are usually considered environmental contaminants, gangrenous necrosis due to *Fusarium acutatum* infection in a patient with diabetic foot ulcerations was reported (Taj-Aldeen, et al., 2006). *Fusarium* is a vascular invasive and disseminated filamentous fungus that responds poorly to antifungal therapy alone. Bone debridement, surgical amputation and adjunct antifungal therapy are usually required for *Fusarium* osteomyelitis and diabetic foot ulcerations (Strom, et al., 2022). Amphotericin B, natamycin and newer azoles such as voriconazole are recommended as the most active antifungal agents for *Fusarium* foot osteomyelitis. Although treatment guidelines do not include this therapy, oral voriconazole is an adequate empirical treatment for *Fusarium* foot osteomyelitis before required susceptibility tests (João, et al., 2021). *Aspergillus conidia* is a ubiquitous commensal fungi in the respiratory tract; however, *Aspergillus* infections in chronic wounds are rare. To date, only a few cases have been reported, mainly focusing on bone and joint ulceration caused by *Aspergillus fumigatus*, *Aspergillus flavus* and *Aspergillus nidulans* in patients with immunosuppression, a variety of underlying chronic diseases or prior surgical interventions. A targeted combination of surgical debridement of necrotic tissue and systemically adapted antimycotic therapy, such as oral voriconazole, is necessary (Koehler, et al., 2014). Several rare *Aspergillus* spp. have been proven to be important in chronic wound infection. *Aspergillus ochraceus* osteomyelitis in a patient with diabetic foot ulcers was reported recently, and the drug tolerance of these uncommon *Aspergillus* spp. deserves attention (Babamahmoodi, et al., 2015). The major feature of chronic wound infections is biofilm development. Biofilms reduce the effectiveness of chronic wound treatment and can increase treatment resistance. Biofilms can also enable bacteria and the host to interact cooperatively or competitively. Biofilms can be found in many human and animal infections, such as cystic fibrosis, otitis media and dental caries (Larsen, et al., 2017; Moser, et al., 2017; Yadav, et al., 2017). In past studies, microbial biofilms were identified in almost 80% of surgical area infections (Romling, et al., 2012).

Fungal disease treatment relies heavily on four classes of antifungal drugs: azoles, polyenes, the pyrimidine analog 5-flucytosine and echinocandins (Robbins, et al., 2017). Amphotericin B, a polyene fungicide possessing broad-spectrum antifungal activities and rare antifungal resistance, has been suggested to be used topically to treat wound infections, but nephrotoxicity and electrolyte abnormalities frequently occurs from long-term administration (Sanchez, et al., 2014). Moreover, treatment failure occurs because of the interplay among antifungal drug properties, underlying host immune defects and fungal characteristics, which include antifungal resistance, antifungal tolerance and diverse cell morphologies (Fisher, et al., 2018). Many fungi have the ability to resist antifungal drugs, such as the ubiquitous mold *Aspergillus fumigatus* and new emerging species, including the yeast *Candida auris* (Rhodes, et al., 2019). The “super fungus” *Candida auris* can invade human localized tissue and cause systemic infection by colonizing in chronic wounds and is known for its pandrug resistance and high lethality (Liu, et al., 2022). In healthcare, drug resistance is significantly exacerbated by biofilms, where the close proximity of pathogens allows facile transfer of resistance-encoding genes (Hetrick, et al., 2009). Severely burned patients usually have many complications, and the most devastating complication is fungal burn wound infections (Palackic, et al., 2021). Fungal burn wound infections can be mistaken for early bacterial burn wound infection and drug-resistant fungi can severely impede wound healing. Thus, developing new antifungal drugs is essential.

In a significant number of chronic wound types, fungal pathogens are a substantial part of the microbial diversity. When wound infections occur, many fungi contribute to the bioburden or biofilm formation of chronic wounds. The diversity of fungal representatives in such wounds is higher than previously reported (Dowd, et al., 2011). When treating chronic wounds, only addressing the bacterial contribution is not sufficient. Ignoring the fungal involvement will result in inadequate treatment. The propensity to overlook the pathogenic potential of *Candida* species likely contributes to inadequate treatment of chronic wound infections, even though it has been isolated on multiple occasions. Specifically, *Candida albicans* and *Candida tropicalis* triggered outbreaks of sternal wound infections in a total of 23 cases (Malani, et al., 2002). Improvements in the healing process will be directly observable if fungal species are targeted using molecular methods (Wolcott, et al., 2010). Dowd et al. reported 3 cases of chronic wounds that showed no improvement after weekly debridement, iodosorb gel and local wound care (Dowd, et al., 2011). Finally, *Candida parapsilosis* and the fungal genera *Fusarium* and *Candida albicans* were found in these three patients’ wounds. Fungal culture is recommended in all patients with nonhealing diabetic wounds, as timely detection of fungal infection and initiation of antifungal treatment in diabetic wounds is significant for better healing and avoiding gangrene or amputations (Kandregula, et al., 2022).

## Diagnostic and therapeutic innovations for chronic wound cases

Currently, chronic wounds are a major healthcare burden worldwide (Roldan-Marin, et al., 2009). Healthy individuals normally heal quickly, while some people, such as patients with diabetes, have factors that can impair the wound healing process. Wound swabs, cleaning, dressing and debridement of the wound bed are the current standard of care for chronic wounds (Dreifke, et al., 2015). In addition, topical antiseptics are recommended as a first-line therapy by recent consensus guidelines for the management and intervention of chronic wounds with multimicrobial biofilms (Schultz, et al., 2017). Diagnosing polymicrobial infections in chronic wound biofilms is difficult. Thus, determining optimal and individual treatment regimens presents significant challenges (Dowd, et al., 2011). When a nonhealing wound occurs, collecting the patient’s medical history and performing a physical examination are essential. Biopsy is helpful if the etiology of the chronic wound is not clear. In recent years, novel and emerging techniques for the detection of *Candida*-related biofilms and the application of antibiofilm agents have been explored to prevent polymicrobial infections and facilitate wound healing (Aswathanarayan, et al., 2022). Due to the diversity of opportunistic fungi in multispecies biofilm infections, some traditional identification techniques have limitations, and quantitative molecular diagnostic methods are sometimes more efficient and effective. Healing rates, prognosis and outcomes are significantly improved by targeted therapies based on molecular diagnostics combined with multimicrobial wound care protocols (Dowd, et al., 2011).

Many therapeutic choices are available (Table 1) (Mihai, et al., 2018). An effective strategy to manage wounds should be preventing and treating infections while also promoting healing and preventing scarring (Mofazzal Jahromi, et al., 2018). The treatment of chronic wounds is mainly based on the TIME concept, which includes tissue debridement, management of infection or inflammation, moisture balance and epithelialization promotion with stem cell therapy (Demidova-Rice, et al., 2012). Conventional methods, such as wound debridement and antibiotics or antimicrobial substances, are usually considered to eradicate wound infection and have limited effectiveness. The risk of therapeutic resistance is the main disadvantage of recurrent antibiotic use in delayed wound healing. Fungal resistance against antifungal agents and uncommon fungal pathogens has been increasing. The most commonly used drugs are voriconazole and amphotericin B (Palackic, et al., 2021). Since 2013, a variety of therapeutic innovations have emerged for chronic wounds, such as the development of bioactive wound dressings, inhibition of MMPs, cytokine-targeted therapy, topical administration of antimicrobial peptides and growth factors, stem cell-based therapy and epithelization promotion through the design of skin equivalents (Krejner, et al., 2015; Anjana, et al., 2017; Annabi, et al., 2017). Cell-based technologies using bone marrow-derived mesenchymal stem cells, adipose-derived cells, epidermal cells and others have been reported to be a new therapeutic strategy for chronic wound healing by enhancing angiogenesis and re-epithelization in preclinical studies (Kucharzewski, et al., 2019). Several new approaches, such as the administration of growth factors and hyperbaric oxygen therapy, have advantages in wound healing. The use of microorganisms (bacteriophages and probiotics), immune-based antimicrobial molecules (some polypeptides, for example, defensins), phototherapy (ultraviolet light, blue light and so on) and photodynamic therapy (Yin, et al., 2013) are also available as alternative treatments. However, the above nontraditional therapies still have not been demonstrated to be effective in treating chronic wounds (Rodriguez-Menocal, et al., 2015; Toyserkani, et al., 2015; Malhotra, et al., 2016; Castellanos, et al., 2017; Lotz, et al., 2017). Some Chinese herbal extracts, such as the methanolic extract of ephedra ciliata, were active against fungal strains (*Candida albicans* and *Aspergillus niger*) *via* downregulation of TNF-α to promote natural wound healing in chronic excision and burn wound healing models (Yaseen, et al., 2020). Wound healing comprises a complex interaction between natural host processes and immunity (Mihai, et al., 2018). A well-defined cascade of these events will finally lead to treatment success.

**TABLE 1:** Wound therapy approaches (Mihai, et al., 2018).

Therapeutic approach	Advantages		Disadvantages		Indication		Example	
Wound debridement	-	Disruption of the mucopolysaccharide matrix. Destabilizes the biofilm's architecture promotes bacterial detachment increases antimicrobial delivery	-	Many promote the inoculation of infection in deeper tissues	-	Chronic wounds	-	Mehanical
	-						-	Enzymatic
	-						-	Biological
	-							
Topical or systemic antibiotic theraphy	-	Low cost	-	Development of antibiotic resistance	-	Acute wounds	-	Fluoroquinolones, tetracycline,rifampin, daptomycin, and vancomycin
	-	Efficient in acute infections	-	Development of antibiotic tolerance in biofilms				
	-	Eradication of planktonic bacteria	-	Microbial inbalance (target both pathogenic and beneficial bacteria)				
	-	Biofilm disruption	-	Multiple side effects				
Topical antiseptic therapy	-	Cytotoxic toward bacteria, fungi, and others microorganisms	-	Might damage host cell and adversely affect wound healing	-	Acute wounds	-	Povidone-iodine, chlorhexidine, hedrogen, peroxide, boric acid, silver Sulfadiazine or nitrate, sodium hypochlorite, mafenide acetate, octenidine dihydrochloride, Ployhexamethylene biguanide (ployhexanide)
					-	Chronic wounds		
Bacteriophage therapy	-	May be efficient against polymicrobial bio film-mediated infections	-	Biological-associated risks	-	Chronic wounds	-	Monophage preparations (staphylococcal bacteriophages, pyocianic bacteriophages)
	-	Very specific for targeted bacterial species	-	Unknown side effects			-	Multiple phage preparations
			-	High production costs				
			-	Applied in low amount as a topic treatment				
Antimicrobial peptides	-	Antimicrobial properties	-	Used in small amounts	-	Chromic Wounds	-	Defensins, magainins, cecropins
			-	Low stability				
			-	Increased volatility				
Probiotic theraphy	-	Accelerates wound healing	-	Insufficient data	-	Chromic Wounds	-	*Lactobacillus* plantarum
	-	Prevents wound colonization, biofilm developent						
	-	Interferes with the quorum sensing of p. aeruginoa						

Recent wound care guidelines emphasize the importance of topical antiseptic therapy and personalized dressings. Although antiseptic agents such as chlorhexidine, povidone-iodine, boric acid and hydrogen peroxide are cytotoxic to bacteria, microorganisms and fungi, they can also damage host cells and have a negative effect on the wound healing process (Mofazzal Jahromi, et al., 2018). Alternative agents are needed due to the increasing incidence of chronic wounds. The ideal therapeutic agent should have a variety of properties, such as immunomodulatory, antimicrobial and regenerative effects (Bertesteanu, et al., 2014). Nanoparticles have shown promise in treating chronic wounds due to their intrinsic antimicrobial properties and drug carrier function (Mihai, et al., 2018). They are unlikely to result in the emergence of resistance due to their ability of delivering only low drug concentrations to infectious agents. They can contribute to the treatment of biofilm formation and modulation of microbial colonization in chronic wounds. Nanocoated wound dressings can contain nanomaterials with antimicrobial properties that can not only target infectious agents but also act as low concentration drugs. Moreover, nanoparticles can act as drug carriers for other antimicrobial agents (such as plant-derived compounds, bacteriophages, antimicrobial peptides), microbiome regulators (probiotics, prebiotics), or agents that can accelerate wound healing (growth factors, stem cells).

## Conclusion and perspectives

Chronic wounds are closely associated with chronic mono- or polymicrobial biofilm infections. They can be characterized by fungal resistance or tolerance to antimicrobials. This review demonstrates that fungi are important contributors to biofilm production and polymicrobial wound infections. Fungi can be recalcitrant to the healing process. Wounds and ulcers can be colonized by host cutaneous, commensal or environmental fungi that trigger local infections, causing fungemia as well as invasive fungal disease. Furthermore, the fungi involved in nonhealing wound-related infections help commensal bacteria resist antibiotics and the host immune response, forcing wounds to become reservoirs for multiresistant species, which are considered a potential key factor in the microbial bioburden of wounds and ulcers. Biofilm establishment is the predominant mechanism of fungal resistance or tolerance to antimicrobials in chronic nonhealing wounds. *Candida albicans* yeast and *Trichophyton rubrum* filamentous fungi are the main fungi in chronic wound infection. Effective treatment of chronic wounds is mainly based on the TIME concept (tissue debridement, management of infection or inflammation, moisture balance and epithelialization promotion with stem cell therapy). New approaches, such as nanomedicine, can influence chronic wound management in the future. The current understanding of fungal diversity in chronic wounds is still limited and requires further investigation and the ability to manage drug-resistant fungal diseases remains a challenge.

## Abbreviations

DFU, diabetic foot ulcer; VLU, venous leg ulcer; ARG, arginase; iNOS, inducible nitric oxide synthase.
